# Fear of Missing Out and its impact: exploring relationships with social media use, psychological well-being, and academic performance among university students

**DOI:** 10.3389/fpsyg.2025.1582572

**Published:** 2025-06-06

**Authors:** Hala Abd Ellatif Elsayed

**Affiliations:** Department of Health Sciences, College of Health and Rehabilitation Sciences, Princess Nourah bint Abdulrahman University, Riyadh, Saudi Arabia

**Keywords:** Fear of Missing Out (FoMO), social media use, psychological well-being, life satisfaction, academic performance, university students

## Abstract

**Background:**

The increasing prevalence of social media has given rise to the Fear of Missing Out (FoMO) phenomenon, characterized by an acute awareness of the rewarding experiences others might be enjoying. FoMO is hypothesized to affect various aspects of individuals’ lives, including their psychological well-being and academic performance. This study explores these relationships among university students, a demographic particularly vulnerable to social media influences.

**Objective:**

To examine the relationships between FoMO, social media use, psychological well-being (as measured by life satisfaction), and academic performance (as measured by GPA) among university students and determine the mediating roles of FoMO and social media use.

**Methods:**

A quantitative, correlational design was employed, collecting data from 521 university students through a cross-sectional survey. Participants completed the Fear of Missing Out Scale (FoMO), the Satisfaction with Life Scale (SWLS), and self-reported GPA. Social media use was assessed via a customized questionnaire. Data were analyzed using SPSS, employing correlation analysis, multiple regression, and mediation analysis.

**Results:**

Findings indicated a strong positive association between FoMO and social media use (*R*^2^ = 0.633, *p* < 0.001), suggesting that higher levels of FoMO lead to increased social media engagement (*B* = 0.834, *p* < 0.001). Contrary to expectations, FoMO was positively rather than negatively correlated with life satisfaction (*R*^2^ = 0.064, *p* < 0.001, *B* = 0.158). Additionally, a strong positive correlation was observed between social media use and academic performance (Spearman’s rho = 0.765, *p* < 0.001). Mediation analyses revealed that FoMO does not significantly mediate the relationship between social media use and life satisfaction, as the indirect effect was not statistically significant (*B* = 0.0785, 95% CI: −0.0164 to 0.1467). Similarly, the non-significant indirect effect indicated that social media use did not mediate the relationship between FoMO and academic performance (*B* = 0.005, 95% CI: −0.0045 to 0.0146). Moderation analyses showed that FoMO moderates the relationship between social media use and life satisfaction, where social media use hurt life satisfaction at low levels of FoMO (*B* = −0.1713, *p* = 0.0001) but had a positive effect at high levels of FoMO (*B* = 0.2848, *p* < 0.0001). This suggests that individuals with high FoMO may derive psychological benefits from social media use. Additionally, results indicated that social media use moderates the relationship between FoMO and academic performance, where FoMO had a significant adverse effect on GPA at low social media use (*B* = −0.030, *p* < 0.0001). Still, this effect became non-significant at high levels of social media use (*B* = 0.0097, *p* = 0.1028). Finally, life satisfaction moderates the relationship between FoMO and social media use, with higher life satisfaction strengthening the positive association between FoMO and social media use (*B* = 0.9277, *p* < 0.0001).

**Conclusion:**

These findings highlight the complex interplay between FoMO, social media use, life satisfaction, and academic performance. While FoMO increases social media engagement, its positive association with life satisfaction contradicts theoretical expectations. These results underscore the importance of considering psychological and social factors when evaluating the impact of social media use among university students.

## 1 Introduction

Social media has shaped how university students connect, communicate, and express themselves in the digital age. While these platforms offer many opportunities, they also foster a phenomenon known as Fear of Missing Out (FoMO)—a form of social anxiety marked by the persistent belief that others are experiencing rewarding events in one’s absence (Przybylski et al., 2013). FoMO is intensified by the curated content on platforms such as Instagram, TikTok, and Snapchat, which constantly exposes students to peers’ highlights and achievements, fueling feelings of exclusion and inadequacy (Jabeen et al., 2023; Tandon et al., 2021).

University students, in particular, are vulnerable to FoMO due to their developmental stage, which places a high value on peer relationships and social validation (O'Connell, 2020; Qutishat and Abu Sharour, 2019). Excessive social media use driven by FoMO has been consistently linked to lower life satisfaction, higher stress levels, and impaired academic performance (Elhai et al., 2020; Alt, 2015). However, the literature on these associations remains divided. Some studies suggest that FoMO may motivate students to engage more actively in academic tasks (Kong et al., 2024), while others report that it undermines self-control and increases procrastination (Manap et al., 2023).

Moreover, much of the existing literature is concentrated in Western contexts, with limited attention to the sociocultural dynamics that may influence the experience and impact of FoMO in Arab populations. As Karimkhan and Chapa (2021) highlight, cultural values such as collectivism, social obligation, and digital connectivity norms can modulate how FoMO manifests and affects well-being. There is a need for region-specific research that contextualizes FoMO within these cultural frameworks.

Theoretically, this study is grounded in two complementary models. The first is Self-Determination Theory (SDT), which posits that psychological well-being depends on the satisfaction of autonomy, competence, and relatedness (Ryan and Deci, 2000). FoMO can be viewed as a consequence of unmet relatedness needs, resulting in compulsive social media engagement and reduced life satisfaction. The second is the I-PACE model (Brand et al., 2016), which explains how personal predispositions (e.g., FoMO), affective responses (e.g., distress), and executive functions (e.g., social media use) interact to shape behavioral outcomes such as academic performance. Recent studies have supported this integrated view. For instance, Soraci et al. (2025) found that FoMO and psychological distress fully mediated the relationship between life satisfaction and problematic technology use. Similarly, Servidio et al. (2024) demonstrated that FoMO influenced problematic social media use through the serial mediation of social comparison and self-esteem.

Despite these developments, few studies have examined how FoMO, social media use, life satisfaction, and academic performance interact within a unified theoretical model, particularly in Arab university contexts. “This study aims to fill this gap by examining the direct, mediating, and moderating relationships among FoMO, social media use, life satisfaction, and academic performance. The goal is to better understand the complex mechanisms through which FoMO influences students’ psychological well-being and academic outcomes. Given the growing reliance on social media among university students, exploring how FoMO influences digital behaviors, emotional outcomes, and academic functioning is crucial, especially in culturally distinct settings.

### 1.1 Social media use and FoMO

Given the increasing integration of social media into university students’ daily routines, exploring the nuanced relationship between social media use and the development of FoMO is essential. Social media platforms facilitate communication and academic collaboration and function as arenas for self-presentation and social comparison (Abbas et al., 2019; Ashraf et al., 2021). Platforms such as Instagram, TikTok, and Snapchat—designed around real-time updates and curated highlights—expose users to a continuous stream of others’ activities and achievements, which can heighten awareness of exclusion and fuel FoMO.

A growing body of research supports the association between FoMO and frequent social media use. Individuals with elevated levels of FoMO are more likely to engage in compulsive checking behaviors, driven by the perceived need to remain socially updated and connected (Przybylski et al., 2013; Fioravanti et al., 2021). However, this relationship is not unidirectional nor uniformly negative. On one hand, active engagement—such as commenting, posting, and messaging—can promote meaningful interaction and help fulfill social needs, thereby potentially reducing FoMO (Mao and Zhang, 2023). On the other hand, passive consumption—characterized by scrolling through others’ content without interaction—has been consistently linked to greater FoMO, increased social comparison, and psychological distress (Jabeen et al., 2023; Mao et al., 2023).

These findings underscore that the style and motivation behind social media use play a critical role in shaping FoMO. Passive use often reinforces upward social comparisons, exacerbating feelings of inadequacy and exclusion. While active use may buffer against these effects, it may still perpetuate FoMO if driven by anxiety or fear of disconnection rather than authentic social connection (Elhai et al., 2020). This highlights the importance of considering users’ internal motivations and cognitive appraisals when examining the psychological effects of social media.

While this section outlines the psychological mechanisms linking social media use to FoMO, it is important to recognize that these effects may not manifest consistently across populations. As discussed in the following section, cultural and social contexts significantly influence how individuals experience and respond to FoMO, reinforcing the need for culturally grounded research.

### 1.2 FoMO and psychological well-being

The relationship between Fear of Missing Out (FoMO) and psychological well-being has garnered substantial scholarly attention, particularly regarding its association with mental health outcomes among young adults. FoMO, marked by persistent anxiety about missing out on rewarding experiences, often compels individuals to maintain constant digital connectivity and social media surveillance (Gupta and Sharma, 2021). This hypervigilance is believed to increase susceptibility to negative emotional states.

Multiple studies have established a negative correlation between FoMO and life satisfaction, indicating that higher levels of FoMO are consistently associated with increased anxiety, depression, and a diminished sense of well-being (Deniz, 2021; Przybylski et al., 2013; Rahmah and Qudsyi, 2024). These outcomes are typically attributed to the pressure to monitor others’ lives and the distress generated by upward social comparisons. Students, in particular, may feel overwhelmed by the constant need to keep up with peers, which can erode self-esteem and psychological balance.

However, the relationship between FoMO and well-being is not always straightforward. For instance, Hetz et al. (2015) observed that while FoMO was widespread among heavy social media users, its direct impact on emotional health was weaker than its influence on academic performance. Similarly, Roberts and David (2020) highlight the dual nature of FoMO, suggesting that it may motivate social engagement that fosters a sense of belonging in some contexts. When digital interactions are perceived as meaningful and reciprocal, FoMO-driven behavior might enhance certain aspects of well-being.

The mode of social media engagement is a key moderating factor in this relationship. A meta-analysis by Gupta and Sharma (2021) demonstrated that passive usage—characterized by passive scrolling and observation—was more strongly linked to increased anxiety and depression, whereas active usage involving direct interaction had a comparatively weaker negative effect (Godard and Holtzman, 2024). These findings underscore the importance of how often individuals use social media and how and why they engage with it.

Furthermore, longitudinal research by Milyavskaya et al. (2018) revealed that FoMO’s detrimental effects may intensify over time, with students reporting greater psychological distress and lower well-being as they progress through their academic journey. These cumulative effects are particularly relevant for university students, who face heightened sensitivity to peer approval and social comparison during this critical developmental period.

In light of these mixed findings, there remains a clear need for continued research, particularly in cultural and educational settings where social validation is emphasized and digital engagement is deeply embedded in student life. Understanding how FoMO operates within such environments is crucial for developing more effective psychological interventions to enhance life satisfaction and emotional resilience.

### 1.3 FoMO and academic performance

The influence of FoMO on academic performance has become an increasingly relevant area of investigation, particularly as students’ academic and digital lives become more intertwined. Several studies suggest that higher levels of FoMO are associated with lower academic achievement, often due to diminished concentration, reduced study time, and difficulty completing academic tasks (Alt, 2015; Qutishat and Abu Sharour, 2019). These challenges are frequently attributed to cognitive overload and attentional fragmentation, as students compulsively monitor social media platforms for fear of missing out on peer activities or information (Al-Furaih and Al-Awidi, 2020).

One frequently cited mechanism underlying this effect is academic procrastination. Manap et al. (2023) found that students with elevated FoMO were more likely to delay academic responsibilities in favor of social media engagement. This behavior increased their vulnerability to internet addiction and impaired academic focus. This suggests that FoMO can divert cognitive and motivational resources from goal-directed academic behaviors, ultimately undermining performance over time.

However, the literature also presents a more complex and sometimes contradictory picture. For instance, Kong et al. (2024) found that while FoMO negatively influenced students’ self-control and focus, it also positively affected learning engagement for some individuals. This paradoxical finding suggests that FoMO may sometimes function as a motivational force, prompting students to remain involved in academic activities to avoid falling behind. Similarly, Ascenzi (2021) reported that although FoMO was linked to increased stress and anxiety, it did not directly predict academic outcomes such as GPA, implying the presence of indirect mechanisms such as self-regulation or emotional coping.

Further complicating the landscape, Mallari et al. (2023) found no significant correlation between FoMO and GPA in their study of college students, suggesting that individual differences in digital coping strategies and academic support may buffer against the adverse effects of FoMO. These inconsistencies point to the need for moderated and mediated models that account for the influence of other psychological and behavioral variables.

These findings indicate that the relationship between FoMO and academic performance is neither linear nor universally negative. Instead, it appears to be shaped by a constellation of factors, including students’ social media behaviors, regulatory capacities, and contextual influences. As with psychological well-being, university students may be particularly vulnerable to the academic consequences of FoMO due to their developmental sensitivity to peer comparison, digital validation, and external expectations.

### 1.4 Theoretical framework: integration of self-determination theory (SDT) and the I-PACE model

The current study draws upon two complementary frameworks: self-determination theory (SDT) and the I-PACE model (Interaction of Person-Affect-Cognition-Execution) to explain the mechanisms underlying the relationship between FoMO, social media use, psychological well-being, and academic performance.

Self-Determination Theory (SDT), developed by Deci and Ryan (1985), posits that optimal psychological functioning relies on fulfilling three basic psychological needs: autonomy, competence, and relatedness. When these needs are thwarted, particularly relatedness, individuals may experience psychological distress and seek compensatory behaviors. In the context of FoMO, unmet social needs lead students to over-engage with social media to maintain connection and gain approval (Przybylski et al., 2013; Elhai et al., 2020). However, this behavior can undermine autonomy and competence by creating dependence on external validation, and may contribute to reduced life satisfaction, academic procrastination, and emotional exhaustion (Alt, 2015; Gupta and Sharma, 2021). This conceptualization is empirically supported by Riordan et al. (2022), who found that FoMO predicted both higher negative and lower positive affect. FoMO moderated the relationship between SNS use and daily affect, exacerbating the emotional toll of online engagement.

In parallel, the I-PACE model (Brand et al., 2016, 2019) provides a dynamic framework for understanding how personal characteristics, such as FoMO, interact with emotional and cognitive responses to influence specific behaviors like social media use. FoMO is viewed as a dispositional factor that activates affective (e.g., anxiety, comparison) and cognitive (e.g., rumination, selective attention) processes linked to behavioral outcomes. The model supports the idea that the relationship between FoMO, social media use, and outcomes such as psychological well-being or academic performance may be explained through indirect pathways. It also considers the role of moderating variables, such as life satisfaction or self-control, which may influence the strength or direction of these associations.

Recent empirical studies grounded in the I-PACE model have demonstrated that mediating mechanisms and moderating variables shape psychological pathways involving FoMO and digital media use. For instance, Servidio et al. (2024) proposed a serial mediation model in which FoMO influenced problematic social media use through social comparison and self-esteem. Their findings underscore the cognitive and emotional mechanisms outlined in the I-PACE model, particularly the role of FoMO in activating self-referential comparisons that erode self-worth and drive maladaptive media use—mechanisms highly relevant to the current study’s focus on university students’ well-being. Similarly, Soraci et al. (2025) demonstrated that both FoMO and psychological distress mediated the relationship between life satisfaction and problematic technology use, supporting the idea that emotional vulnerability and unmet psychological needs serve as indirect routes through which FoMO exerts its effects. In line with the current model, their results emphasize how lower life satisfaction can exacerbate FoMO and related digital compulsions, further linking subjective well-being to behavioral outcomes. Additionally, Gezgin (2025) found that mindfulness moderated the relationship between FoMO and problematic media use, highlighting how individual-level psychological resources can buffer the adverse effects of FoMO. This supports the broader conceptualization that factors such as life satisfaction or patterns of social media use may similarly moderate the strength and direction of the associations between FoMO, well-being, and academic performance in university students.

The Riordan et al. (2022) study further strengthens this integrated framework by empirically confirming that trait FoMO amplifies the emotional effects of SNS use, specifically, greater SNS use was associated with increased negative affect only among individuals with high FoMO. Their findings underscore SDT’s relevance, suggesting that unmet psychological needs (especially relatedness) contribute to maladaptive social media engagement patterns. Moreover, the daily-diary methodology used in their study offers strong ecological validity and supports the dynamic person-affect-cognition-execution processes outlined in the I-PACE model.

By integrating SDT and I-PACE, the current study conceptualizes FoMO as both a dispositional predictor and a moderator, social media use as both a mediator and a moderator, and life satisfaction and academic performance as key outcomes.

### 1.5 Specification of variable roles within the theoretical framework

Grounded in the theoretical principles of Self-Determination Theory (SDT) and the I-PACE model, the current study proposes a conceptual model that captures both the motivational foundations and behavioral mechanisms of FoMO-related outcomes. Each variable in the model plays a distinct role, informed by these theoretical perspectives.

FoMO is conceptualized as the primary independent variable at the model’s core. According to SDT, FoMO reflects an unmet need for relatedness, which may result in heightened anxiety and compensatory behaviors, such as excessive digital engagement, that undermine autonomy and competence. From the I-PACE perspective, FoMO is a dispositional factor that triggers emotional (e.g., stress, insecurity) and cognitive (e.g., comparison, vigilance) processes, influencing downstream behaviors and outcomes.

Social media use serves a dual role in the model. First, it is positioned as a mediating variable that explains how FoMO translates into negative psychological and academic outcomes. I-PACE supports this pathway by suggesting that maladaptive digital behavior stems from cognitive-affective activation driven by personal vulnerabilities such as FoMO. Second, social media use functions as a moderator, potentially intensifying the negative impact of FoMO on academic performance when its use becomes compulsive or unregulated.

Life satisfaction is conceptualized as an outcome (dependent variable) and a moderating variable. Within SDT, life satisfaction is seen as a reflection of successful need fulfillment. It is hypothesized to buffer the effects of FoMO on social media use, such that students with high life satisfaction may be less likely to engage in compensatory or compulsive digital behaviors, even when experiencing FoMO. Furthermore, FoMO is expected to negatively predict life satisfaction, as persistent social comparison and unmet social needs diminish well-being.

Academic performance, measured via GPA, represents the second primary dependent variable. Both SDT and I-PACE suggest that the psychological strain and attentional fragmentation caused by FoMO and excessive social media use can impair self-regulation, concentration, and motivation, leading to suboptimal academic outcomes. However, this relationship may vary depending on individual and contextual factors, hence the inclusion of moderation paths.

Finally, FoMO is examined as a moderator in the relationship between social media use and life satisfaction. Based on I-PACE, individuals high in FoMO may be more vulnerable to the negative emotional impact of digital overuse, thus amplifying the detrimental consequences of social media engagement on well-being.

### 1.6 Study objectives

Building on Self-Determination Theory (SDT) and the I-PACE model, this study explores the interrelated pathways through which Fear of Missing Out (FoMO) influences university students’ psychological well-being and academic performance. Specifically, it seeks to:

1. Examine the direct relationships between FoMO, social media use, life satisfaction, and academic performance.
2. Test whether social media use and FoMO function as mediators between key constructs.
3. Investigate the moderating effects of FoMO, social media use, and life satisfaction in shaping psychological and academic outcomes.
4. Contextualize these dynamics within an Arab university setting, addressing the cultural factors that may influence these relationships.

### 1.7 Hypotheses

#### 1.7.1 Direct effects

*H1*: Higher levels of FoMO are positively associated with greater social media use among university students.

*H2*: Higher levels of FoMO are negatively associated with life satisfaction.

*H3*: Social media use is negatively associated with academic performance.

#### 1.7.2 Mediation effects

*H4*: FoMO mediates the relationship between social media use and life satisfaction.

*H5*: Social media use mediates the relationship between FoMO and academic performance.

#### 1.7.3 Moderation effects

*H6*: FoMO moderates the relationship between social media use and life satisfaction, such that higher FoMO amplifies the negative association.

*H7*: Social media use moderates the relationship between FoMO and academic performance, intensifying its adverse effect.

*H8*: Life satisfaction moderates the relationship between FoMO and social media use, weakening the positive association among students with higher life satisfaction.

## 2 Method

### 2.1 Research design

This study employed a quantitative, cross-sectional correlational design to examine the direct, mediating, and moderating relationships among Fear of Missing Out (FoMO), social media use, life satisfaction, and academic performance. Given the proposed model’s complexity, which involves mediating and moderating effects, multiple regression and PROCESS macro analyses were employed to assess direct, indirect, and interaction effects. These methods allow for the testing of hypothesized pathways, including mediation and moderation, while accounting for the influence of covariates and providing estimates of effect sizes.

### 2.2 Participants

A total of 521 university students (aged 18–25 years) enrolled in undergraduate programs across various universities in Saudi Arabia participated in the study. Participants were recruited through convenience sampling with the support of participating universities, which distributed the survey via official university email channels. Participation was voluntary and anonymous, and informed consent was obtained before data collection.

Participants met the following inclusion criteria: (a) enrollment in a university program, (b) age between 18 and 25 years, and (c) active use of social media. Individuals who were not currently enrolled in a university or who did not actively use social media were excluded. The final sample was diverse in academic discipline and represented a range of Saudi higher education institutions.

## 3 Data collection procedures

### 3.1 Survey administration

The survey was hosted on a secure online platform and distributed electronically. Respondents first reviewed an informed consent form, outlining the studys’ purpose, confidentiality, and voluntary participation. The survey took approximately 15–20 min to complete. The survey distribution was facilitated through the universities’ official communication systems, specifically via student university email accounts. This process was enabled through a formal service known as “Tasheel Mohimmat Baheth” (Researcher Task Facilitation), which supports academic research by allowing institutions to assist in data collection efforts.

### 3.2 Ethical considerations

Ethical approval was obtained from the university’s ethics committee. Participants were assured of the confidentiality and anonymity of their responses and informed of their right to withdraw at any point without penalty.

## 4 Measures

Validated Arabic versions of all measures were used, with no additional translation required. Psychometric properties were reassessed in the current sample using Cronbach’s alpha for internal consistency and confirmatory factor analysis (CFA) for structural validity. Exploratory factor analysis (EFA) was also conducted during the preliminary assessment.

### 4.1 Fear of Missing Out (FoMO) scale

Fear of Missing Out (FoMO) was assessed using the FoMO Scale, initially developed by Przybylski et al. (2013). The scale consists of 10 items designed to measure individuals’ concerns about missing out on rewarding experiences that others may be having. Each item is rated on a 5-point Likert scale, ranging from 1 (Not at all true of me) to 5 (Extremely accurate of me), with higher scores indicating greater fear of missing out. The original English version of the scale has demonstrated high internal consistency (Cronbach’s *α* = 0.87 to 0.90) and strong construct validity, as it correlates positively with social media engagement and negatively with psychological need satisfaction (Przybylski et al., 2013).

The Arabic version of the FoMO Scale, translated and validated Al-Menayes (2020b), was used for this study. The translation process followed a standardized forward-backward translation method, ensuring linguistic and conceptual equivalence between the original English version and the Arabic adaptation. The Arabic version has been psychometrically validated, demonstrating strong internal consistency (Cronbach’s *α* = 0.82–0.89) and maintaining the same unidimensional structure as the original. The scale has also been used in prior studies to examine the relationship between FoMO and social media use in Arabic-speaking populations.

To ensure the reliability of the Arabic-translated FoMO Scale in this study, Cronbach’s alpha was calculated, with α > 0.70 considered acceptable. Confirmatory factor analysis (CFA) was also conducted to verify the scale’s factorial structure within the study population.

### 4.2 Satisfaction with life scale (SWLS)

Life satisfaction was assessed using the Satisfaction with Life Scale (SWLS), initially developed by Diener et al. (1985). The SWLS is a widely used measure designed to assess individuals’ cognitive evaluation of their overall life satisfaction. It consists of five items, each rated on a 7-point Likert scale, ranging from 1 (strongly disagree) to 7 (strongly agree), with higher scores indicating greater life satisfaction (Diener et al., 1985). The scale has demonstrated strong psychometric properties across different populations, making it one of the most reliable tools for measuring subjective well-being.

The Arabic version of the SWLS was used for this study, translated and validated by Taisir Abdallah (1998). The translation process followed a standardized forward-backward translation method, ensuring linguistic and conceptual equivalence between the original English version and the Arabic adaptation. The validation study was conducted on 864 Arabic-speaking university students, confirming that the Arabic version retains the same factorial structure as the original and demonstrating strong internal consistency (Cronbach’s *α* = 0.79) and test–retest reliability (*r* = 0.83) (Abdallah, 1998). The scale also showed significant correlations with other validated psychological measures, further supporting its construct and concurrent validity.

The scoring system for the SWLS follows the guidelines provided by Diener (2006), which classifies life satisfaction levels into six categories:

31–35: Extremely satisfied.
26–30: Satisfied.
21–25: Slightly satisfied.
15–19: Slightly dissatisfied.
10–14: Dissatisfied.
5–9: Extremely dissatisfied (Diener, 2006).

To ensure the reliability of the Arabic-translated SWLS in this study, Cronbach’s alpha was calculated, with *α* > 0.70 considered acceptable. Additionally, confirmatory factor analysis (CFA) was conducted to verify the factorial structure of the scale within the study population. The psychometric properties observed in this study align with previous research, further supporting the validity and reliability of the Arabic version of the SWLS for assessing life satisfaction among Arabic-speaking participants.

### 4.3 Social media use measurement

Social media use was assessed using the Social Media Addiction Scale (SMAS), an adaptation of Young’s Internet Addiction Test (IAT) designed to measure problematic social media usage. The original IAT, developed by Young (1998, 2011), consists of 20 items assessing excessive Internet use, neglect of responsibilities, and emotional attachment to online activities. To create the SMAS, the word “Internet” was replaced with “social media,” ensuring that the items specifically targeted social media-related behaviors rather than general Internet usage (Young, 1998, 2011).

The Arabic version of the SMAS was developed and validated by Al-Menayes (2015, 2020a). Psychometric evaluation of the scale demonstrated good internal consistency (Cronbach’s *α* = 0.75–0.86) and a three-factor structure, covering social consequences, time displacement, and compulsive tendencies (Al-Menayes, 2015). Exploratory factor analysis (EFA) and confirmatory factor analysis (CFA) supported the construct validity of the scale, and concurrent validity was confirmed through correlations with the Social Media Engagement Questionnaire (SMEQ) (Al-Menayes, 2015).

The original IAT has been widely validated across different populations. For example, Widyanto and McMurran (2004) conducted a factor analysis of the IAT, identifying six distinct factors related to salience: excessive use, neglect of work, anticipation, self-control, and neglect of social life. These findings support the structural validity of the IAT as a foundation for measuring online behaviors. Hawi (2013) also validated an Arabic version of the IAT, demonstrating strong internal consistency and factorial validity, further supporting its applicability in Arabic-speaking populations. Given that the SMAS is derived from the IAT, these findings reinforce its psychometric credibility (Hawi, 2013; Widyanto and McMurran, 2004).

For this study, the Arabic-translated version of the SMAS was used. The scale underwent a forward-backward translation process to ensure linguistic and conceptual equivalence, following best practices for adapting psychological measures. The reliability of the Arabic version in the current sample was assessed using Cronbach’s alpha, with *α* > 0.70 considered acceptable. Additionally, CFA was conducted to verify the factorial structure of the scale in the study population.

### 4.4 Academic performance

The students’ academic performance was measured using self-reported Grade Point Average (GPA) on a 4-point scale. Self-reported GPA is commonly used in psychological research as an indicator of academic achievement and has been shown to correlate strongly with official academic records (Kuncel et al., 2005). While self-reported GPA may be subject to potential reporting biases, prior research suggests that it remains a valid and reliable measure of academic performance in survey-based studies.

## 5 Statistical analysis

The statistical analysis was conducted using SPSS software and the PROCESS Macro. Simple linear regression was employed to examine the relationships between variables and to test the proposed hypotheses. Spearman’s rho correlation was used to assess the relationship between GPA and social media addiction (SMA). The PROCESS Macro (Model 1 and Model 4) was utilized to test for mediation and moderation effects. The bootstrapping method was applied in cases where regression was conducted for GPA, as it is an ordinal variable, to ensure robust and reliable estimates. The reliability of the scales was assessed using Cronbach’s alpha coefficient, which demonstrated good reliability: 0.878 for the SMA scale, 0.915 for the FoMO scale, and 0.849 for the Satisfaction with Life Scale (SWLS). A confidence interval of 95% and a margin of error of 0.05 were considered statistically significant throughout the analysis.

## 6 Results

### 6.1 Participants characteristics

A total of 521 university students participated in the study. As shown in Tables 1, 2, the sample was predominantly female (67.4%) and Saudi (93.3%), with most aged between 18 and 24 years (90%). Most were single (82.1%) and reported a monthly family income below 5,000 SAR (70.4%). Additionally, 74.3% of participants indicated they were not taking psychological medications. Regarding academic background, most students were from Health Colleges (46.1%) and Science Colleges (23.4%). Participants were fairly distributed across academic levels, with the highest concentration in Level 1 (31.5%) and Level 2 (28.8%). GPA distribution showed that over half (55.1%) had an excellent GPA, while 29.6% reported very good performance.

**Table 1:** Demographic characteristics of the study participants.

Demographic characteristics	Frequency	%
Gender		
Male	170	32.6
Female	351	67.4
Nationality		
Saudi	486	93.3
Non-Saudi	35	6.7
Age		
18–24	469	90.0
25–34	45	8.6
35–44	7	1.3
Marital status		
Single	428	82.1
Married	62	11.9
married and have children	19	3.6
Not married, divorced, widowed, and have children	12	2.3
Income		
Less than 5,000 SAR/month	367	70.4
5,000–10,000 SAR/month	74	14.2
10,000–20,000 SAR/month	54	10.4
20,000–30,000 SAR/month	18	3.5
more than 30000SAR/month	8	1.5
Psycho medication		
Yes	134	25.7
No	387	74.3

**Table 2:** Education characteristics of the study participants.

Education characteristics	Frequency	%
College		
Health Colleges (Medicine, Dentistry, Health and Rehabilitation etc)	240	46.1
Scientific Colleges (College of Sciences, College of Engineering, College of Computer and Information Sciences)	122	23.4
Humanities Colleges (College of Education, College of Arts, College of Law, …etc)	90	17.3
Institutes (Institute of Arabic Language, Institute of English Language)	21	4.0
Applied College	48	9.2
Education level		
level1	164	31.5
level2	150	28.8
level3	63	12.1
level4	32	6.1
level5	38	7.3
Diploma/master/PhD	74	14.2
GPA		
Failed	2	0.4
Accepted	16	3.1
Good	62	11.9
Very good	154	29.6
Excellent	287	55.1

### 6.2 Main analyses

The results of the simple linear regression analyses are presented in Table 3. FoMO significantly predicted social media addiction (SMA), explaining 63.3% of its variance (*R*^2^ = 0.633, *p* < 0.001), with a strong regression coefficient (*B* = 0.834), indicating that higher levels of FoMO were associated with increased social media use. Contrary to the study’s expectations, FoMO was also found to have a positive association with life satisfaction (SWLS), although the effect size was relatively small (*R*^2^ = 0.064, *B* = 0.158, *p* < 0.001). In addition, a significant and unexpected positive correlation emerged between SMA and GPA (Spearman’s *ρ* = 0.765, *p* < 0.001), as shown in Table 4. This finding challenges prevailing assumptions that social media engagement necessarily impairs academic performance, suggesting instead a more complex relationship that may involve compensatory or adaptive behaviors among students.

**Table 3:** Results of simple linear regression.

Independent	Adjusted R^2^	*B*	*F*	Sig	Durbin-Watson
FoMO--------SMA	0.633	0.834	896.312	0.000*	1.807
FoMO--------SWLA	0.064	0.158	35.764	0.000*	1.827

**Table 4:** Correlation between SMA and GPA.

	Correlation coefficient	Sig. (2-tailed)	*N*
SMA
	0.000*		
		0.765	
		521

*Correlation is significant at the 0.05 level (2-tailed).

### 6.3 Mediation analyses

**Figure 1:**
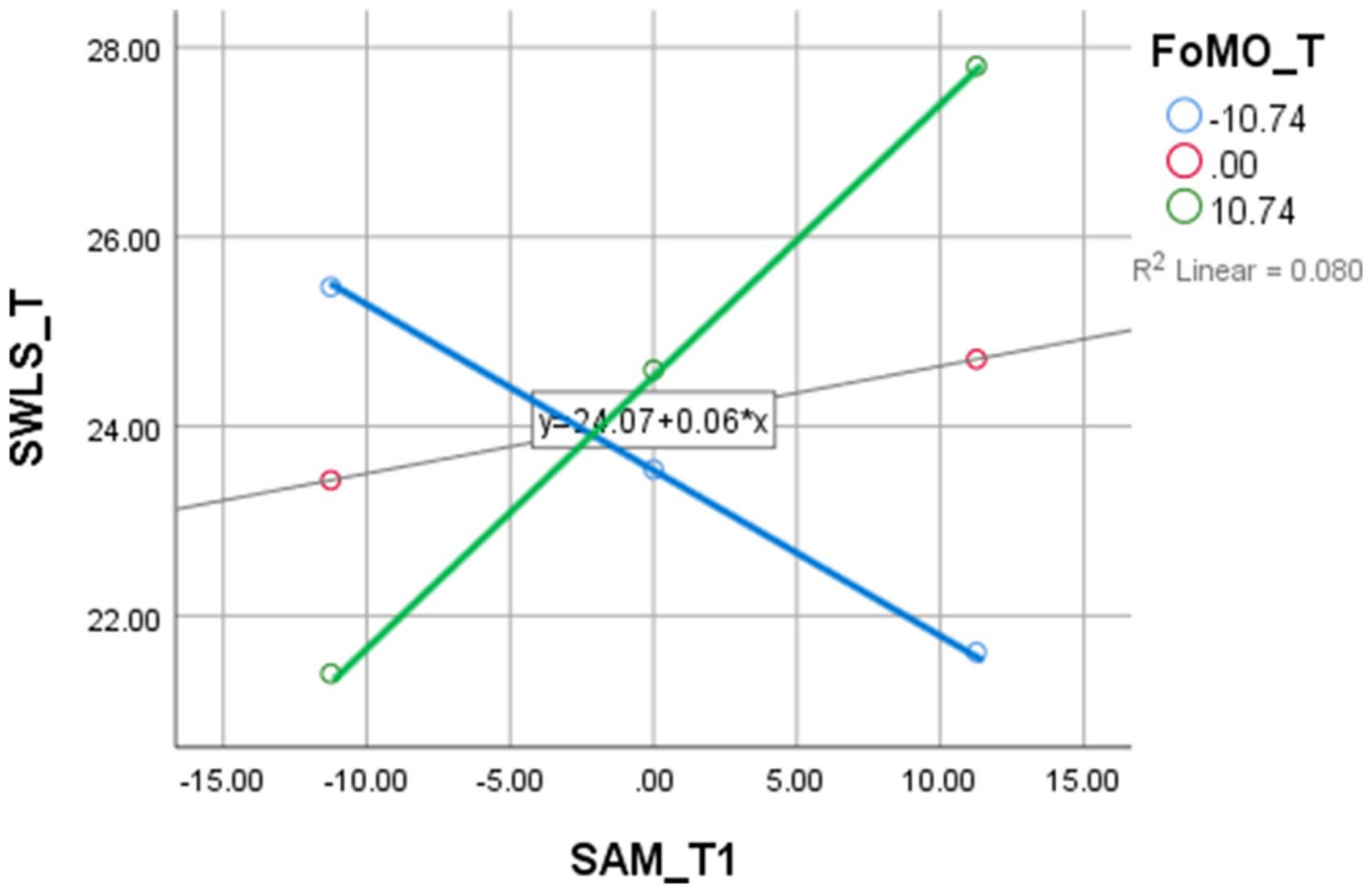
Interaction effect of Social Media Addiction (SMA) and Fear of Missing Out (FoMO) on Life Satisfaction (SWLS). The figure illustrates the moderating effect of FoMO on the relationship between SMA and SWLS. As FoMO increases, the association between SMA and life satisfaction becomes more positive, indicating that individuals with higher FoMO may experience greater life satisfaction through increased social media engagement.

Two mediation models were tested to investigate the indirect effects among the key study variables. The first model examined FoMO as a mediator in the relationship between SMA and SWLS (Table 5). The analysis revealed that SMA significantly predicted FoMO (*R*^2^ = 0.6333, *p* < 0.001), and the total effect of SMA on SWLS was statistically significant (*B* = 0.1436, *p* < 0.001). However, the indirect effect of SMA on SWLS through FoMO was insignificant, with a confidence interval that included zero (CI: −0.0053 to 0.1574). These results indicate that FoMO is not a significant mediator between SMA and life satisfaction. As shown in Figure 1, FoMO significantly predicted social media use.

**Table 5:** Mediation effect of FoMO in the relationship between social media use and life satisfaction.

Relationship	Total effect	Direct effect	Indirect effect	Confidence interval		Conclusion
				Lower Bound	Upper Bound	
SMA- < FoMO - < SWLS	0.1436 < 0.001	0.0652 0.1170	0.0785	−0.0164	0.1467	No medication role

In the second model, SMA was tested as a mediator between FoMO and GPA (Table 6). As expected, FoMO significantly predicted SMA (*B* = 0.8345, *p* < 0.001). However, neither the direct effect of FoMO on GPA nor the indirect effect through SMA was statistically significant. The confidence interval for the indirect effect (CI: −0.0045 to 0.0147) also included zero, confirming the absence of a mediation effect. Accordingly, SMA does not appear to mediate the relationship between FoMO and academic performance.

**Table 6:** The medication effect of social media use in the relationship between FoMO and academic performance.

Relationship	Total effect	Direct effect	Indirect effect	95% bootstrap CI		Conclusion
				Lower Bound	Upper Bound	
FoMO - < SMA - < GPA	0.0060 0.2629	−0.0056 0.3192	0.005	−0.0045	0.0146	No medication role

### 6.4 Moderation analyses

**Figure 2:**
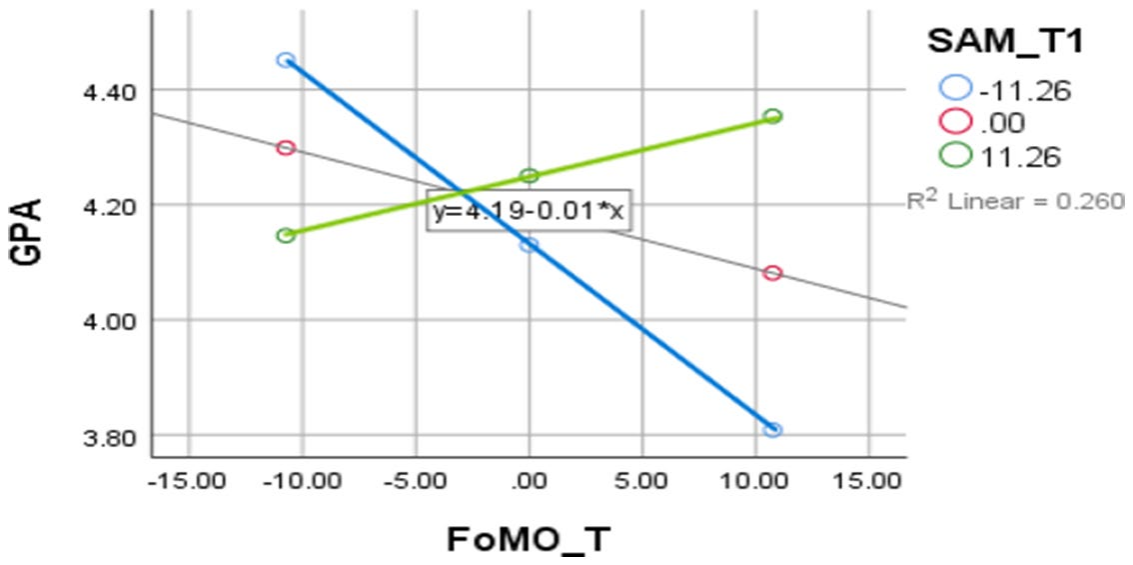
Moderating effect of Social Media Addiction (SMA) on the relationship between Fear of Missing Out (FoMO) and Academic Performance (GPA). The graph shows that the relationship between FoMO and GPA is moderated by SMA levels. At higher levels of SMA, the association between FoMO and GPA is more positive, suggesting that social media engagement may serve as a compensatory mechanism for academic performance among those with high FoMO.

To further explore interaction effects, three moderation models were tested. The first model examined FoMO as a moderator of the relationship between SMA and SWLS (Table 7). The interaction term was significant (*B* = 0.0212, *p* < 0.001), indicating that the strength and direction of the relationship between social media use and life satisfaction varied depending on levels of FoMO. Specifically, higher FoMO amplified the positive association between SMA and SWLS, suggesting that individuals with elevated FoMO may derive more satisfaction from social media engagement. The mediation model is illustrated in Figure 2.

**Table 7:** The moderating effect of FoMO on the relationship between social media use and life satisfaction.

Relationship	*R* ^2^	Beta	SE	*T*	*p*-value	Conclusion
Moderating effect (SMA*FoMO)- < SWLA	0.243	0.01212	0.0020	10.88	0.000	FoMO has a moderating effect

*Correlation is significant at the 0.05 level (2-tailed).

The second model tested SMA as a moderator of the relationship between FoMO and GPA (Table 8). The interaction effect was significant (*B* = 0.0018, *p* < 0.001), showing that the relationship between FoMO and GPA depended on the level of social media use. At low levels of SMA, FoMO negatively predicted GPA. At the same time, this effect was non-significant at high levels of SMA, indicating a buffering or compensatory effect of social media use on academic outcomes among high-FoMO individuals.

**Table 8:** The moderating effect of SMA in the relationship between FoMO use and GPA.

Relationship	*R* ^2^	Beta	SE	*T*	*p*-value	Conclusion
Moderating effect (FoMO *SMA)- < GPA	0.0783	0.0018	0.0003	1.03	0.000	SMA has a moderating effect

*Correlation is significant at the 0.05 level (2-tailed).

**Figure 3:**
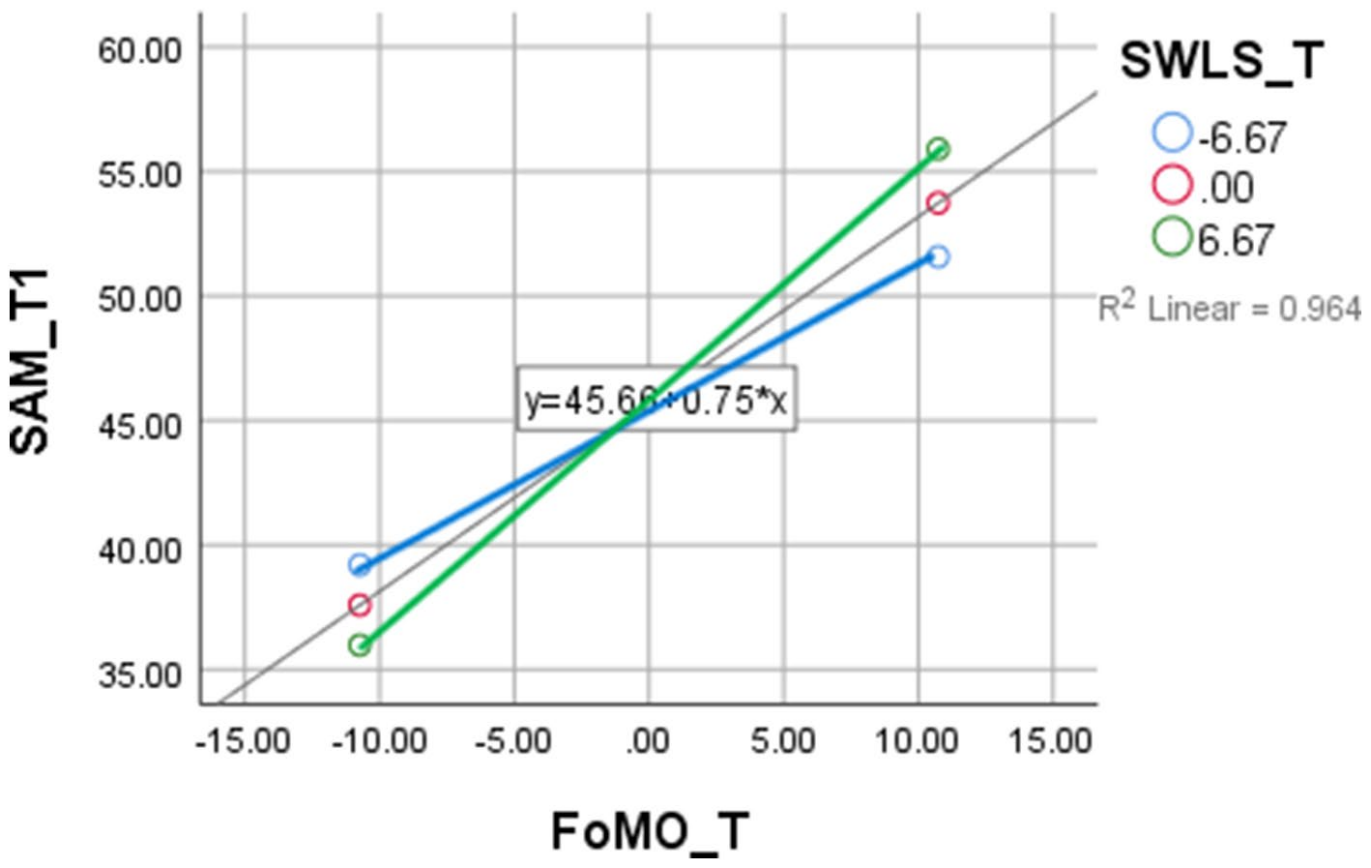
Moderating effect of Life Satisfaction (SWLS) on the relationship between Social Media Addiction (SMA) and Fear of Missing Out (FoMO). This figure displays how higher levels of life satisfaction strengthen the positive relationship between SMA and FoMO, indicating that individuals with greater satisfaction may perceive social media use as more rewarding, potentially reinforcing FoMO.

**Figure 4:**
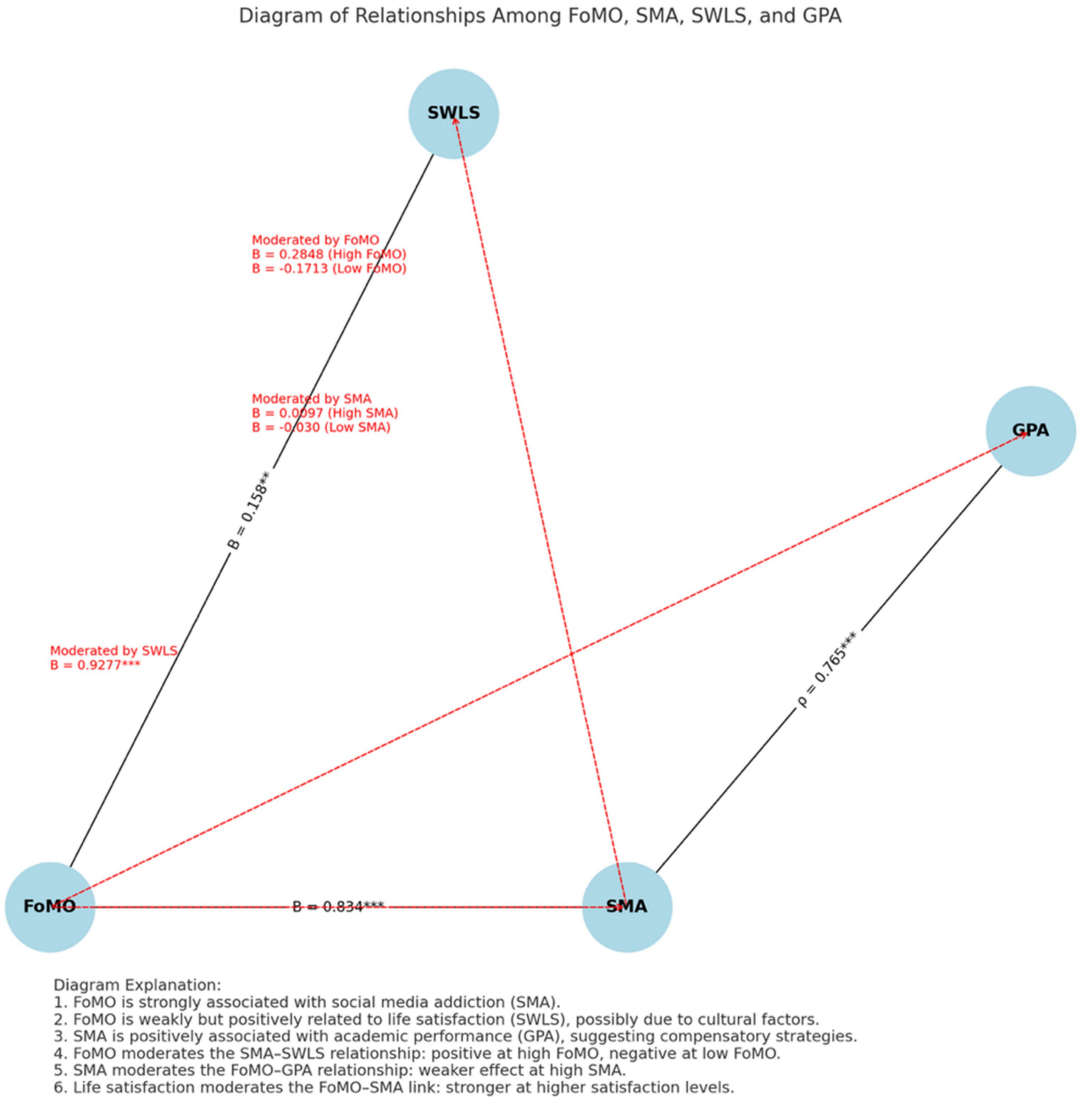
Final moderated model illustrating significant direct and interaction effects among FoMO, SMA, SWLS, and GPA. This conceptual model summarizes all significant paths: FoMO positively predicts SMA and weakly predicts SWLS; SMA is positively associated with GPA. Moderation effects include: (1) FoMO moderates the SMA–SWLS link, (2) SMA moderates the FoMO–GPA link, and (3) SWLS moderates the FoMO–SMA link.

The third model examined whether life satisfaction moderated the relationship between FoMO and SMA (Table 9). The results indicated a significant interaction (*B* = 0.0263, *p* < 0.001), with higher life satisfaction intensifying the positive relationship between FoMO and social media use. This suggests that individuals with high life satisfaction and FoMO engage more heavily with social media, potentially perceiving it as a positive or rewarding activity. Figure 3 demonstrates the moderating role of FoMO in the SMA -SWLS relationship. As depicted in Figure 4, life satisfaction moderates the link between FoMO and social media use.

**Table 9:** Moderation effect of Life satisfaction in the relationship between FoMO and SMA.

Relationship	*R* ^2^	Beta	SE	*t*	*p*-value	Conclusion
Moderating effect (FoMO *SWLS)- < SMA	0.6634	0.0263	0.004	6.61	< 0.0001	SWLS has a moderating effect

*Correlation is significant at the 0.05 level (2-tailed).

## 7 Discussion

This study provides nuanced insights into the interrelationships among Fear of Missing Out (FoMO), social media addiction (SMA), life satisfaction (SWLS), and academic performance (GPA) in a sample of Saudi university students. Guided by the I-PACE model, the findings affirm several theoretical assumptions while challenging others, particularly in the cultural context.

### 7.1 FoMO and social media use

As expected, FoMO was strongly and positively associated with social media addiction (SMA) (*B* = 0.834, *p* < 0.001; *R*^2^ = 0.633). This finding is consistent with previous studies (Przybylski et al., 2013; Fioravanti et al., 2021; Mao and Zhang, 2023), reinforcing the notion that individuals experiencing heightened FoMO are more inclined to engage compulsively with social media. The I-PACE model supports this association, positing that cognitive and affective vulnerabilities, such as the anxiety of being excluded, act as motivational drivers for excessive digital use. This robust relationship observed in our sample underscores the relevance of FoMO as a central predictor of problematic social media behaviors among university students.

### 7.2 FoMO and psychological well-being

Contrary to the prevailing literature, which generally associates FoMO with reduced psychological well-being (Gupta and Sharma, 2021; Sommantico et al., 2023), our findings revealed a small but statistically significant positive association between FoMO and life satisfaction (*B* = 0.158, *p* < 0.01). One possible explanation for this discrepancy is cultural: in collectivist societies such as Saudi Arabia, heightened social awareness and engagement, often driven by FoMO, may enhance an individual’s sense of inclusion and satisfaction.

Moreover, the moderating effect of FoMO on the SMA–SWLS relationship was significant. Specifically, the relationship between SMA and life satisfaction became more positive at higher levels of FoMO (*B* = 0.2848 at high FoMO, *B* = −0.1713 at low FoMO), suggesting that individuals with higher FoMO may derive greater life satisfaction from their social media engagement. These findings imply that FoMO, in this cultural context, may act less as a source of psychological strain and more as a motivator for socially rewarding behaviors.

### 7.3 FoMO and academic performance

Although FoMO was positively associated with SMA, the present findings did not support a statistically significant direct relationship between FoMO and GPA (*p* > 0.05). This result contrasts with prior research (Alt, 2015; Qutishat and Abu Sharour, 2019), which emphasized FoMO’s negative academic impact due to distraction and poor attention control.

Additionally, social media use was positively correlated with GPA (*ρ* = 0.765, *p* < 0.001), contrary to initial expectations of a negative impact. This surprising finding aligns with Ascenzi (2021), who noted that students with higher digital engagement may compensate for distraction by developing adaptive coping mechanisms such as time management and multitasking skills.

The moderation analysis further showed that SMA moderated the FoMO–GPA relationship, with a weaker negative association between FoMO and GPA at high levels of SMA (*B* = −0.030 at low SMA; *B* = 0.0097 at high SMA, n.s.). This suggests that highly active students on social media may buffer the academic consequences of FoMO more effectively.

### 7.4 Mediation and moderation effects

Although the I-PACE model (Brand et al., 2016, 2019) and prior empirical research (e.g., Elhai et al., 2020) conceptualize social media use as a potential mediating pathway linking FoMO to both psychological and academic outcomes, this assumption was not supported in the current study. The mediation analyses yielded nonsignificant results for both hypothesized pathways. Specifically, social media use did not significantly mediate the relationship between FoMO and life satisfaction (*B* = 0.0785, 95% CI [−0.0164, 0.1467], *n.s.*), nor did it mediate the relationship between FoMO and academic performance (B = 0.005, 95% CI [−0.0045, 0.0146], *n.s.*). One possible explanation is that the direct relationship between FoMO and life satisfaction (*B* = 0.158, *p* < 0.01) may have been strong enough to render the indirect effect redundant. Additionally, the positive association between social media use and academic performance (*ρ* = 0.765, *p* < 0.001) contradicts the assumption that social media behaviors undermine students’ academic functioning.

In the cultural context of Saudi Arabia, social media use may serve more adaptive roles, such as fostering academic collaboration or social inclusion, rather than contributing to psychological or academic decline. This may weaken the indirect effects of FoMO on both life satisfaction and academic performance through social media use. These findings point to the importance of exploring alternative mediating mechanisms, such as emotion regulation, digital literacy, or self-control, that may more effectively explain the complex pathways from FoMO to life outcomes across diverse cultural settings.

According to the I-PACE model (Brand et al., 2016; 2019), person-related variables such as FoMO interact with affective and cognitive states (e.g., life satisfaction) and behavioral patterns (e.g., social media use) to produce variable outcomes. Thus, this model provides a theoretical basis for exploring not only direct and indirect effects but also conditional (moderated) relationships among these constructs.

The moderation analyses yielded significant results that highlight the conditional nature of the relationships under study. First, FoMO significantly moderated the association between social media use and life satisfaction, such that the relationship became more positive at higher levels of FoMO (*B* = 0.2848 at high FoMO vs. *B* = −0.1713 at low FoMO). This finding suggests that students with heightened FoMO may experience greater psychological rewards from engaging with social media, perhaps due to the increased sense of connection and social inclusion that such platforms provide.

Second, social media use moderated the relationship between FoMO and academic performance. Although this interaction did not yield a statistically significant effect at high levels of social media use (*B* = 0.0097, n.s.), the association was more negative at low levels (*B* = −0.030), suggesting that students who are less active on social media may be more vulnerable to the potential academic costs of FoMO. This may reflect the development of adaptive strategies among high-engagement users, such as multitasking or time management, which buffer against academic decline.

Finally, life satisfaction moderated the relationship between FoMO and social media use. Specifically, higher levels of life satisfaction intensified the association between FoMO and social media use (*B* = 0.9277, *p* < 0.001), indicating that students who are more satisfied with their lives may feel more confident or justified in using social media as a means of maintaining social involvement driven by FoMO. Together, these findings underscore the dynamic and interactive nature of FoMO’s influence, which depends not only on behavioral patterns but also on the psychological context in which social media is used.

### 7.5 Conceptual model

The diagram illustrates the complex relationships among FoMO, social media addiction (SMA), life satisfaction (SWLS), and academic performance (GPA) based on the study’s findings. A strong positive relationship was observed between FoMO and SMA, indicating that students who experience higher levels of FoMO are more likely to engage in compulsive social media use. Interestingly, FoMO also showed a weak but statistically significant positive association with life satisfaction, which may be influenced by cultural factors in the Saudi context—where social connectedness, even when driven by FoMO, might enhance subjective well-being. At the same time, SMA was positively correlated with GPA—an unexpected finding that challenges the dominant narrative of social media being purely distracting. This result suggests that students who are more digitally engaged may compensate through adaptive strategies such as time management or multitasking, thereby maintaining or even enhancing their academic performance.

Moderation analyses revealed significant conditional effects. FoMO moderated the relationship between SMA and life satisfaction: students with higher FoMO experienced greater life satisfaction from their social media engagement (*B* = 0.2848), whereas those with lower FoMO showed a negative association (*B* = −0.1713). In turn, SMA moderated the relationship between FoMO and GPA. At high levels of SMA, the association between FoMO and GPA became negligible (*B* = 0.0097). In contrast, at low levels of SMA, the association was more negative (*B* = −0.030), suggesting that active social media users may buffer the academic consequences of FoMO. Finally, life satisfaction moderated the relationship between FoMO and SMA, such that students with higher life satisfaction exhibited a stronger link between FoMO and social media use (*B* = 0.9277). These findings underscore the importance of considering the interactive and cultural context in which FoMO operates and highlight the need to move beyond simple correlational assumptions to better understand these nuanced dynamics.

### 7.6 Cultural considerations

The role of cultural context in shaping FoMO experiences cannot be overlooked. Social belonging and communal engagement are highly valued in cultures such as Saudi Arabia. Unlike in Western societies, where FoMO may lead to loneliness and dissatisfaction, this study’s context might drive greater social participation, thus enhancing life satisfaction. Karimkhan and Chapa (2021) support this perspective, emphasizing the importance of cultural factors in understanding FoMO-related behaviors.

### 7.7 Limitations and future directions

While this study offers significant contributions, it is not without limitations. The cross-sectional design limits the ability to draw causal conclusions. Future research should employ longitudinal designs to examine how FoMO and social media use evolve. Additionally, relying on self-reported measures may introduce bias, emphasizing the need for objective assessments of social media usage and academic performance. Future studies should also explore additional moderating variables, such as personality traits and coping strategies, to provide a more comprehensive understanding of these relationships.

## 8 Conclusion

In conclusion, this study contributes to the growing body of research on FoMO by offering a culturally grounded analysis of its associations with social media use, life satisfaction, and academic performance among Saudi university students. Guided by the I-PACE model, the findings reveal a strong direct link between FoMO and social media use, while challenging assumptions about its negative impact on well-being and academic outcomes. The absence of significant mediation effects suggests that alternative mechanisms—such as emotion regulation or digital literacy—may better explain the pathways from FoMO to key outcomes. Importantly, the significant moderation effects highlight that these relationships are conditional and influenced by psychological and behavioral factors, underscoring the interactive and dynamic nature of digital engagement. These insights call for more culturally sensitive models that consider the role of context, coping strategies, and individual differences when examining the implications of FoMO in digitally connected societies.

## Data Availability

The raw data supporting the conclusions of this article will be made available by the authors, without undue reservation.
